# Precision Modeling of Alzheimer’s Disease via Integrative Pathway Activity Analysis across Human Brains and Cellular Models for Discovering Translatable Therapeutic Targets

**DOI:** 10.1002/alz70859_106163

**Published:** 2025-12-26

**Authors:** Pourya Naderi Yeganeh, Sang Su Kwak, Mehdi Jorfi, Weiming Xia, Rudolph E. Tanzi, Doo Yeon Kim, Winston Hide

**Affiliations:** ^1^ Beth Israel Deaconess Medical Center, Boston, MA USA; ^2^ Harvard Medical School, Boston, MA USA; ^3^ Massachusetts General Hospital, Charlestown, MA USA; ^4^ Boston University Chobanian & Avedisian School of Medicine, Boston, MA USA

## Abstract

**Background:**

Successful Alzheimer’s disease (AD) interventions in preclinical models often fail in human trials. While preclinical models offer insights into AD mechanisms, there is no systematic approach to verify whether preclinical target mechanisms retain therapeutic relevance in humans. Bridging this preclinical‐to‐clinical translational gap accelerates therapeutic development by precisely addressing whether failures are due to testing ineffective drugs, targeting the wrong mechanism, or relying on unrepresentative models.

**Method:**

We have developed a novel bioinformatics platform, named Integrative Pathway Activity Analysis (IPAA), that maps pathway activity from omics data. IPAA precisely captures the degree to which disease functions in models match those in human brains and prioritizes targetable pathways in the most representative models. We assessed the mechanistic similarities between the transcriptomes of three AD brain regions and multiple 2D/3D human AD cellular models to define targetable functions. We performed phosphoproteomics analysis and compared pathway activity changes with transcriptomic findings. Top pathways were pharmacologically evaluated for their impact on AD pathology in 3D models.

**Result:**

IPAA found high correlation of pathway dysregulation between brain regions (r=0.84, temporal cortex and parahippocampal gyrus), suggesting IPAA’s ability to detect conserved AD functions. IPAA found 83 dysregulated transcriptomic pathways shared between AD brains and a 3D model with a high Amyloid‐beta (Aβ) 42/40 ratio. Shared dysregulated pathways included p38 MAPK, YAP1/TAZ, E‐cadherin, CDC20, and APC/C, which were confirmed at the protein level. Elevated active p38 MAPK was observed in the 3D models, human AD brains, and 5XFAD mice, localized to presynaptic dystrophic neurites. Phosphoproteomic analysis confirmed an increase in p38 MAPK substrate phosphorylation driven by Aβ42 accumulation. Targeting p38 MAPK with a clinical p38α/β MAPK inhibitor (Losmapimod)– which has not been tested for AD– significantly reduced Aβ‐induced tau, Aβ accumulation, neuronal loss, and microglial activation in 3D models and human microglia. We further found that MAPK‐activated protein kinase 2 (MK2) plays crucial roles in mediating Aβ‐induced tau pathology.

**Conclusion:**

IPAA enables rapid preclinical assessment of target pathways with confidence for impact on AD pathology prior to clinical trials. Our findings highlight the critical role of protein kinase networks, particularly the p38 MAPK‐MK2 axis, in driving AD pathology in humans.

